# A randomized, double-blind, placebo-controlled trial of soluble corn fiber supplementation for children with asthma

**DOI:** 10.3389/falgy.2025.1707834

**Published:** 2026-01-21

**Authors:** Matthew A. Rank, Daisy L. Barroso, Kathryn A. Conn, Melissa Pecak, Matthew Scandura, Allyson H. Hirsch, Haiwei Gu, Corrie M. Whisner, Heather Shearer, Darcy Johnson, Natalia Argel, Cindy S. Bauer, Sophia N. Williams, Benjamin L. Wright, James Woodward, Emily K. Cope

**Affiliations:** 1 Division of Allergy, Asthma, and Clinical Immunology, Mayo Clinic in Arizona, Scottsdale, AZ, United States; 2 Division of Pulmonology, Phoenix Children’s, Phoenix, AZ, United States; 3 Department of Biological Sciences, The Pathogen and Microbiome Institute, Northern Arizona University, Flagstaff, AZ, United States; 4 Division of Allergy and Immunology, Phoenix Children’s, Phoenix, AZ, United States; 5 Department of Molecular & Biomedical Sciences, University of Maine, Orono, ME, United States; 6 Informuta, Inc., San Diego, CA, United States; 7 College of Health Solutions, Arizona State University, Phoenix, AZ, United States; 8 Center for Health Through Microbiomes, The Biodesign Institute, Arizona State University, Tempe, AZ, United States; 9 Department of Pharmacy, Phoenix Children’s, Phoenix, AZ, United States

**Keywords:** asthma, fiber, gut microbiome-lung axis, microbiome, short chain fatty acids

## Abstract

**Introduction:**

Asthma is a multifactorial disease influenced by genetic and environmental factors, including diet. The gut microbiome contributes to airway inflammation via the gut-lung axis, partly through production of short chain fatty acids (SCFAs) from bacterial fermentation of dietary fiber. We hypothesized that dietary fiber supplementation could modulate the gut microbiome and increase SCFAs in children with asthma.

**Methods:**

This is a double-blind, placebo-controlled trial of children who were randomized to consume 12 g of soluble corn fiber (SCF) as a supplement to their usual daily diet (50% the recommended daily fiber intake) or placebo for 4–6 weeks (clinicaltrials.gov NCT03673618). Dietary surveys, asthma symptom questionnaires, fecal, blood and nasal samples were collected before and after the intervention period to quantify fiber intake, asthma control, nasal and gut microbiome, and serum short chain fatty acids (SCFAs).

**Results:**

Of the 20 children enrolled, 15 completed the intervention with an average adherence rate of 83%. SCFA concentrations and gut microbiome changes varied by individual and treatment group. No significant differences in gut or nasal alpha or beta diversity were observed between groups post-intervention. However, differential abundance analysis showed a trend toward increased *Bifidobacterium* in the SCF group compared to placebo (ANCOM-BC *p* = 0.0004, FDR *q* = 0.073).

**Discussion:**

Supplementation of 50% of recommended daily fiber intake had minimal impact on asthma symptoms, the microbiome, or SCFA levels. Future studies should consider higher fiber doses, different fiber types, or targeting individuals with low baseline fiber intake to account for observed variability in microbiome and SCFA responses.

**Clinical Trial Registration:**

https://clinicaltrials.gov/study/NCT03673618, identifier NCT03673618.

## Introduction

Asthma is a complex inflammatory airways disease that affects approximately 300 million people in the world, and nearly 24.8 million persons in the US (7.7% of the US population) (1). In industrialized populations, such as the United States, asthma prevalence has increased over the past 40 years, from ∼3% in 1981 to 7.7% in 2021 (2–5). The rapid emergence of asthma as one of the most common chronic inflammatory diseases in the past 40 years points to environmental changes, rather than genetic, as primarily responsible for the trend. Environmental changes due to modern lifestyle are strongly implicated as important factors driving asthma incidence and severity. One potential lifestyle factor is the rise in consumption of the Western (industrialized) diet, which is high in refined carbohydrates, fats, and salt, and markedly low in dietary fiber (6–8). A recent analysis of 23,042 participants in the National Health and Nutrition Examination Survey (NHANES) highlights the importance of dietary quality in respiratory health. In this study, higher dietary quality scores were associated with reduced respiratory disease; consumption of fiber-rich fruits and vegetables emerged as one of the most significant food groups associated with respiratory health (8). Products of microbial fermentation of dietary fibers are suggested to modulate components of the immune system through epigenetic mechanisms (9) and signaling through G protein-coupled receptors (GPCRs; 10, 11) in ways that may be clinically beneficial in asthma, obesity, cancer, and diabetes (12–15). However, the underlying mechanisms and clinical significance have yet to be fully understood. Because of this, there is increasing interest in exploring dietary components as modifiable factors influencing respiratory health. Mechanistically, dietary fibers are not digested by human intestinal enzymes resulting in their transport into the large intestine and colon. Many fibers are fermented by the gut microbiota into short chain fatty acids (SCFAs), including acetate, propionate, and butyrate. SCFAs are immunomodulatory and, in preclinical studies, are important metabolites that reduce type-2 inflammation, which is common in childhood asthma (11, 16–18).

Dietary fibers are a diverse group of carbohydrates found in plant-based foods and play a crucial role in shaping the gut microbiome. However, their effects can vary significantly depending on the type and dose consumed. Different fibers, such as soluble fibers like inulin, pectin, soluble corn fiber, resistant starches, and insoluble fibers like cellulose, provide distinct substrates for microbial fermentation, leading to varied production of SCFAs and other metabolites. Although most prebiotic fiber interventions have been performed in healthy populations, the taxa and metabolites found enriched are associated with immune modulation (19–22). In many studies, supplementation with the soluble fibers inulin and galacto-oligosaccharides results in enrichment of fiber-fermenting *Bifidobacterium* and *Lactobacilli* (23–25). Similarly, a whole food based, fiber-enriched diet (40–50 g/day) increased the relative abundance of *Bifidobacterium* and *Lactobacillus* in the gut microbiome in a healthy adult population (26). Species of *Bifidobacterium* and *Lactobacillus* have been studied for their potential as probiotics in gastrointestinal inflammatory diseases and asthma with differing outcomes (27–30). Early life intervention over the first 6 months of life with *Lactobacillus rhamnosis* GG did not have a strong effect on delaying or preventing development of asthma or eczema at 2 years of age (27). However, an 8-week intervention with *Ligilolactobacillus salivarius* and *Bifidobacterium breve* in a large cohort of pediatric asthma patients (*n* = 442, ages 4–10) reduced the number of asthma exacerbations when compared to the group receiving placebo (28). The impact of a prebiotic dietary fiber intervention in asthma is less well studied; understanding the nuanced interaction between dietary fiber, gut microbiome composition, and health status is essential for developing targeted dietary interventions to promote optimal health in the gut and peripheral organs (23–25).

Manipulation of the gut microbiome-airway axis to improve asthma or type 2 inflammation has been previously tested in several preclinical and clinical studies. Gut microbial metabolism of prebiotic dietary fiber into SCFAs may have local and peripheral effects. Locally, in the gut, these promote expansion of CD4 + FOXP3+ regulatory T cells [Treg; (9, 31)] or peripherally to reduce activation of T2-polarizing dendritic cells DCs in the lung (11, 32), or by enhancing airway Treg cells through epigenetic modifications (33). Another study demonstrated microbiome-driven allergic lung inflammation was reduced by SCFAs as demonstrated in 2 airway inflammation models (11, 33). Trials in human participants with asthma are limited, and demonstrate conflicting results. In one, a single dose of fiber (3.5 g inulin) + probiotics via yogurt in patients with asthma reduced airway inflammatory markers compared to controls when measured 4 h post ingestion (32). A 4-week placebo-controlled trial with synbiotics (galactooligosaccharide and fructooligosaccharide fibers + *Bflidobacterium*) demonstrated a beneficial effect on some airway outcomes (systemic T2 cytokine production and peak expiratory flow rates) but not others (bronchial inflammation and airway hyperresponsiveness; (34). However, a 7-day randomized three way cross-over trial of fiber (inulin 12 g/day), inulin + probiotic, or placebo did not show significant changes in the gut microbiome, changes in plasma short chain fatty acids (SCFAs), or changes in patient-reported or objective asthma measures between groups (35). *Post-hoc* analyses suggested possible relationships between fiber group and asthma symptoms and sputum inflammatory markers. The authors speculated that the timing of the SCFA measurement (12 h post fasting) may have missed peak plasma SCFA levels (35). Finally, a trial of increasing fruits and vegetables in children with asthma similarly did not reduce asthma exacerbations (36). Taken together, animal models and early human studies exploring the relationship between fiber intake and airway inflammation in asthma suggest an association, but the mechanistic links, the necessary fiber dose, the type of fiber, the individuals who may best respond, and the outcomes most likely to be impacted all remain unclear.

Here, we performed a pilot clinical trial of dietary modulation of gut microbiota via a prebiotic soluble corn fiber. The primary hypothesis tested in this study is that children with asthma who take a prebiotic soluble corn fiber supplement will have higher plasma SCFAs than children who take placebo. The secondary hypotheses are that the changes in SCFA plasma concentrations will be linked to background fiber dietary intake, changes in the gut microbiome, and decreased T2 inflammation in the airways. We demonstrate highly individualized responses to fiber intake, which can inform future studies evaluating the link between fiber intake and peripheral inflammation in a complex human population.

## Materials and methods

### Ethics

Participants were enrolled at the Phoenix Children's Hospital (PCH) general pulmonary and Severe Asthma Clinic by Allergy/Immunology and Pulmonology physicians (MR, CB, BW, and JW). The study was reviewed and approved by PCH Institutional Review Board (IRB #18-121). The study was registered on clinicaltrials.gov NCT03673618 (Title: Soluble Corn Fiber Supplementation for Asthma). Participants provided written (8–17 years old) or oral (6–7 years old) assent along with parental consent. The first patient enrolled for this study was 10/30/2019.

### Population

Inclusion criteria were children 6–17 years old, stable asthma (no systemic corticosteroid in the past month), no recent hospital visits, and supporting evidence of probable type 2 (T2) inflammation based on either an exhaled nitric oxide of 50 parts per billion or higher or a history of positive allergy tests (skin prick of specific IgE) to at least 1 environmental allergen. Exclusion criteria were other pulmonary disease (e.g., cystic fibrosis, bronchiectasis), planned change in asthma medications in the next 6 weeks, and a special/unique diet (e.g., low-FODMAP for irritable bowel syndrome as assessed by the investigators). No participants were excluded based on diet. To recruit individuals, we assessed the Severe Asthma Clinic schedule each week and approached potential participants and their guardians. We also received referrals from the allergy clinical at Phoenix Children's. The number of participants screened was not recorded.

### Study design

**Figure 1:**
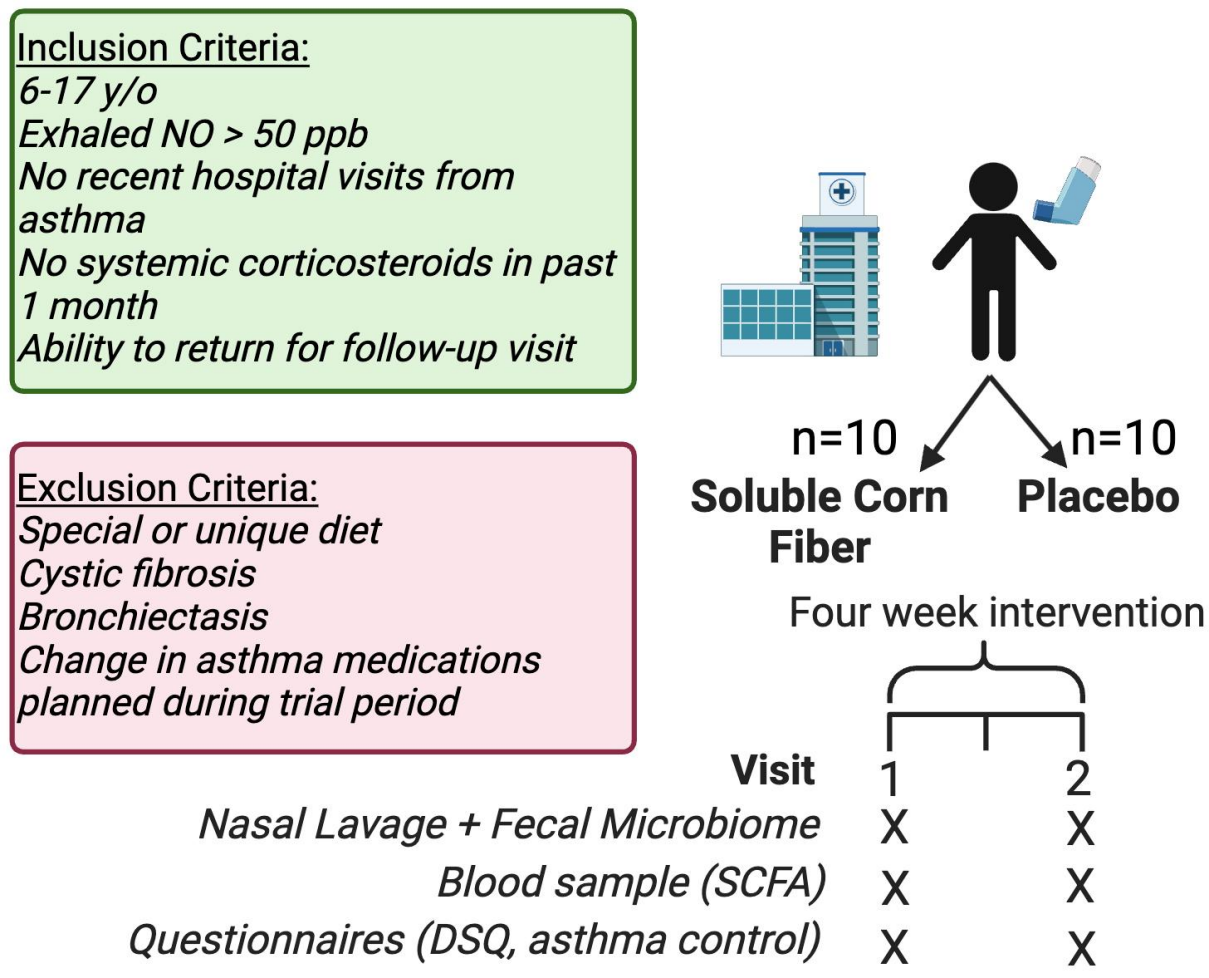
Overview of study design. We performed a double-blind, randomized controlled trial of fiber or placebo supplementation in children with asthma. Twenty participants were enrolled, and 15 completed the 4–6-week intervention. Figure created with biorender.com.

This is a randomized, placebo-controlled, and double-blinded clinical trial. Participants were randomly assigned (1:1) by the study pharmacist (DJ) using a random number generator to ingest PROMITOR (Tate & Lyle, London, UK) soluble corn fiber (81% fiber, 12 g/day of fiber, <2% sugar, caloric content 1.2 kcal/g) in a fruit-flavored beverage of their choosing or placebo (maltodextrin) in a similar fruit-flavored beverage for 4–6 weeks (Figure 1). We chose to supplement half of the daily recommended intake to ensure tolerability in this pilot study. We instructed participants to add the SCF or placebo powder into a flavored beverage to mask the potential sweet taste of the maltodextrin placebo. The fiber and maltodextrin were supplied in similar appearing, slightly off-white powders. To supply 12 g of fiber from 81% soluble corn fiber, the pharmacist weighed out 14.8 g of powder for each dose that was prescribed and packaged it in identical clear sachets. The clear sachets were then packaged in identical light protect baggies that would provide additional blinding for the study staff. The pharmacist supplied the intervention directly to the participants, concealing group assignment, provided education on its administration, and maintained the blinded code. For administration, the participants were instructed to daily add one fiber or placebo packet of powder to 20–30 mL (∼1 ounce) of fruit-flavored beverage or until the powder is dissolved and to drink the entire solution immediately. If powder remained, they were instructed to rinse the cup with more liquid and drink the solution. The remaining members of the study team, participants, and their families/guardians were all unaware of the intervention assignment until after the study was completed and data analyzed. Adherence to the study protocol was measured by the number of dispensed packets returned used or unused.

### Outcomes

Fecal samples, nasal lavage, blood samples, and two questionnaires were collected pre- and post-intervention. Blood collection was not timed relative to the most recent supplement intake. Fecal samples were collected using a home collection fecal kit; participants were asked to swab used toilet paper and mail samples to Northern Arizona University. A clinical research nurse instructed participants on how to collect nasal samples using 15 mL single-use lavage kits (NeilMed). Participants were instructed to insert the nasal aspirator into one nostril, tilt their head, and gently collect the wash from the opposite nostril into a provided 50-mL conical tube. Serum was isolated immediately after blood was drawn and shipped to NAU on dry ice. Participants were not fasting nor were they advised to consume their fiber supplement or placebo timed to the blood draw. Thus, blood collection was not timed relative to fiber intake. We did not record when participants last consumed the fiber. Serum was stored at −80 °C at PCH until shipped in batches on dry ice to NAU.

Participants were asked to complete two questionnaires: the Asthma Control Questionnaire (ACQ) and the Dietary Screener Questionnaire (DSQ). The ACQ is a 7-question, patient-completed questionnaire validated for children ages 6 and up (37) ACQ is scored by taking the total score and dividing by 7, with a range of 0.0–6.0. An ACQ score of <1.5 suggests that the child's asthma is in good control, and a change of >0.5 from measurement to measurement suggests a change that is significant (i.e., the minimal important difference). The National Cancer Institute's Dietary Screener Questionnaire (DSQ) from the National Health and Nutrition Examination Survey (NHANES) is a 26-item questionnaire used to assess food and drink intake in the past 30 days (38). This tool measures dietary consumption across several food groups including fruits and vegetables, processed foods, added sugar, dietary fiber, and whole grains.

An exploratory outcome for this study was whether fiber supplementation could impact airway inflammation. Nasal wash was collected quantification of nasal cytokines in addition to microbiome sequencing, but not analyzed for inflammation due to specimen temperature compromise, which occurred after 16S rRNA gene sequencing.

### DNA extraction and 16S rRNA gene sequencing

DNA and RNA were extracted in parallel using the Qiagen AllPrep Kit (Qiagen) with an additional mechanical lysis. The barcoded primers 515F/806R were used to target the V4 region of the 16S rRNA gene as previously described (39). Each polymerase chain reaction (PCR) contained 2.5 µL of PCR buffer (TaKaRa, 10x concentration, 1x final), 1 µL of the Golay barcode tagged forward primer (10 µM concentration, 0.4 µM final), 1 µL of bovine serum albumin (ThermoFisher, 20 mg/mL concentration, 0.56 mg/µL final), 2 µL of dNTP mix (TaKaRa, 2.5 mM concentration, 200 µM final), 0.125 µL of HotStart ExTaq (TaKaRa, 5 U/µL, 0.625 U/µL final), 1 µL reverse primer (10 µM concentration, 0.4 µM final). For fecal samples, 1 µL of template DNA was included. For nasal lavage, 3 µL of template DNA was included. All PCR reactions were filled to a total 25 µL with PCR grade water (Sigma-Aldrich) then placed on a ThermalCycler. A 30-cycle PCR was used for fecal samples and a 35-cycle PCR was used for the low bacterial burden nasal lavage samples. ThermalCycler conditions were as follows, 98 °C denaturing step for 2 min, 30 (fecal swab) or 35 (nasal lavage) cycles of 98 °C for 20 s, 50 °C for 30 s, and 72 °C for 45 s, a final step of 72 °C for 10 min. PCR was performed in triplicate for each sample and an additional negative control was included for each barcoded primer. A post-PCR quality control step was performed using a 2% agarose gel (ThermoFisher). Extraction blank controls were processed through the 16S PCR identically to tissue samples. Barcoded primer NTCs controls were carried through the agarose gel step. PCR products were quantified using the Qubit dsDNA High Sensitivity Kit (ThermoFisher) and the Qubit fluorometer 4. PCR products were pooled at equimolar concentrations of 50 ng. Quality of the pool was assessed with the Bioanalyzer DNA 1000 chip (Agilent Technologies) then combined with 1% PhiX. A total of two pools were sequenced on the Illumina MiSeq using the 600-cycle MiSeq Reagent Kit V3 (Illumina). Each pool contained three identical samples and a mock community to assess sequencing run variations.

### Quantification of SCFAs via gas chromatography—mass spectrometry

After collection, blood was centrifuged at 4 °C for 15 min at 2,000 × *g* to allow for serum separation. Serum was transferred to a fresh tube, stored at −80 °C until shipment to NAU on dry ice. At NAU, serum was placed at −70 °C until shipment to the Arizona State University (ASU) Metabolomics Lab for gas chromatography-mass spectrometry (GC-MS) measurement of SCFAs (40). For SCFA extraction, 20 µL of serum was mixed with 30 µL of 0.1 M NaOH. 20 µL of 200 µM hexanoic acid-6,6,6-d_3_ was added as an internal standard. To each mixture, 430 µL of MeOH was added and vortexed for 10 s, then placed at −20 °C for 20 min. Samples were centrifuged at 14,000 rpm for 10 min at 4 °C and 450 µL were moved to a new tube. Samples were left to dry under vacuum at 37 °C for 120 min using a CentriVap Concentrator (Labconco, Fort Scott, KS). Derivatization proceeded by adding 40 µL of methoxyamine hydrochloride solution in pyridine (MeOX, 20 mg/mL) and incubating at 60 °C for 90 min. After incubation, 60 µL of N-tert-butyldimethylsilyl-N-methyltrifluoroacetamide (MTBSTFA) was added and incubated for an additional 30 min at 60 °C. The sample was vortexed and centrifuged at 14,000 rpm for 10 min. 70 µL of supernatant was transferred to a new glass vial to be used on the Agilent 7820A gas chromatography system coupled to an Agilent 5977B mass spectrometer (Agilent Technologies, Santa Clara, CA). Separation occurred with a HP-5 ms capillary column coated with 5% phenyl-95% methylpolysiloxane (30 m × 250 µm i.d., 0.25 µm film thickness, Agilent Technologies). We injected 1 µL of each sample and set the solvent delay time to 5 min. The initial oven temperature was held at 60 °C for 1 min, ramped up to 325 °C at a rate of 10 °C/min, and finally held at 325 °C for 10 min. Helium was used as the carrier gas at a constant flow rate of 20 mL/min through the column. The temperature of the front inlet was at 250 °C, the transfer line was 290 °C, and the electron impact (EI) ion source was 230 °C. The electron energy was −70 eV. The mass spectral data were collected in the full scan mode (m/z 30–600). The retention time and quantification mass for each SCFA were determined using respective chemical standards. The concentrations of SCFAs in biological samples were calculated using calibration curves constructed from the corresponding standards. Detection limits were at the magnitude of µg/mL for each SCFA.

### Microbiome bioinformatics

Paired-end sequences were analyzed using Quantitative Insights into Microbial Ecology 2 (QIIME 2) version 2019.10 (41). Demultiplexed sequences were denoised, trimmed at a median *Q* score of 30, and grouped into amplicon sequence variants (ASVs) using dada2 (42). A phylogenetic tree was generated using q2-fragment-insertion (43). Taxonomy was assigned using a Naive Bayes classifier trained on Greengenes 13_8 99% reference OTUs (44). The resulting table of ASV counts on a per sample basis (i.e., the ASV table) was used to compute alpha and beta diversity measures with q2-diversity. Faith's Phylogenetic Diversity was used to measure alpha diversity (within-sample diversity). Unweighted and Weighted Unifrac (45) were used to measure beta diversity and Principal Coordinates (PCoA) plots were visualized using Emperor (46). PERMANOVA was used to determine changes in community composition based on the beta diversity distance matrix. Kruskal Wallis was used to determine changes in alpha diversity across categorical variables, and a nonparametric Mann Whitney test was used to compare delta values of Unifrac (weighted and unweighted), Shannon, and Richness between SCF and placebo groups. To determine whether taxa were differentially abundant at Visit 2, we used Analysis of Compositions of Microbiomes with Bias Correction (ANCOMBC; 47) and corrected for multiple comparisons using the FDR method.

### Statistical analysis

The primary outcome was a change in the SCFA plasma levels (propionate, butyrate, and acetate) as measured by gas chromatography comparing 4-week levels in the placebo and intervention groups. An unpaired Student's *t*-test was used to compare delta values of each SCFA between SCF and placebo groups. *P* values were corrected for multiple comparisons using FDR, using the *p*.adjust package in R. ACQ scores were compared pre- and post-intervention using a paired Student's *t*-test. Changes in dietary variables from the DSQ (e.g., fiber, sugar, protein, carbohydrates) were compared using a Student's *t*-test on the delta values from Visit 2-Visit 1 across groups. Microbiome outcomes were analyzed as described above (*Microbiome Bioinformatics*). A sample size of 20 participants (randomized 1:1) was selected based on the literature describing a significant change in airway inflammatory markers in 17 adults with asthma after a single fiber-rich meal (32). Graphpad Prism v. 10.2.3 was used to generate plots.

#### Data deposition

Raw sequence data were deposited in NIH Sequence Read Archive (SRA) accession number: PRJNA1258290.

#### Role of funders

The Flinn Foundation and the NIH/NIMHD (SHERC U54MD012388) had no role in the study design, interpretation of results, or reporting of outcomes.

## Results

### Clinical trial summary and outcomes

**Figure 2:**
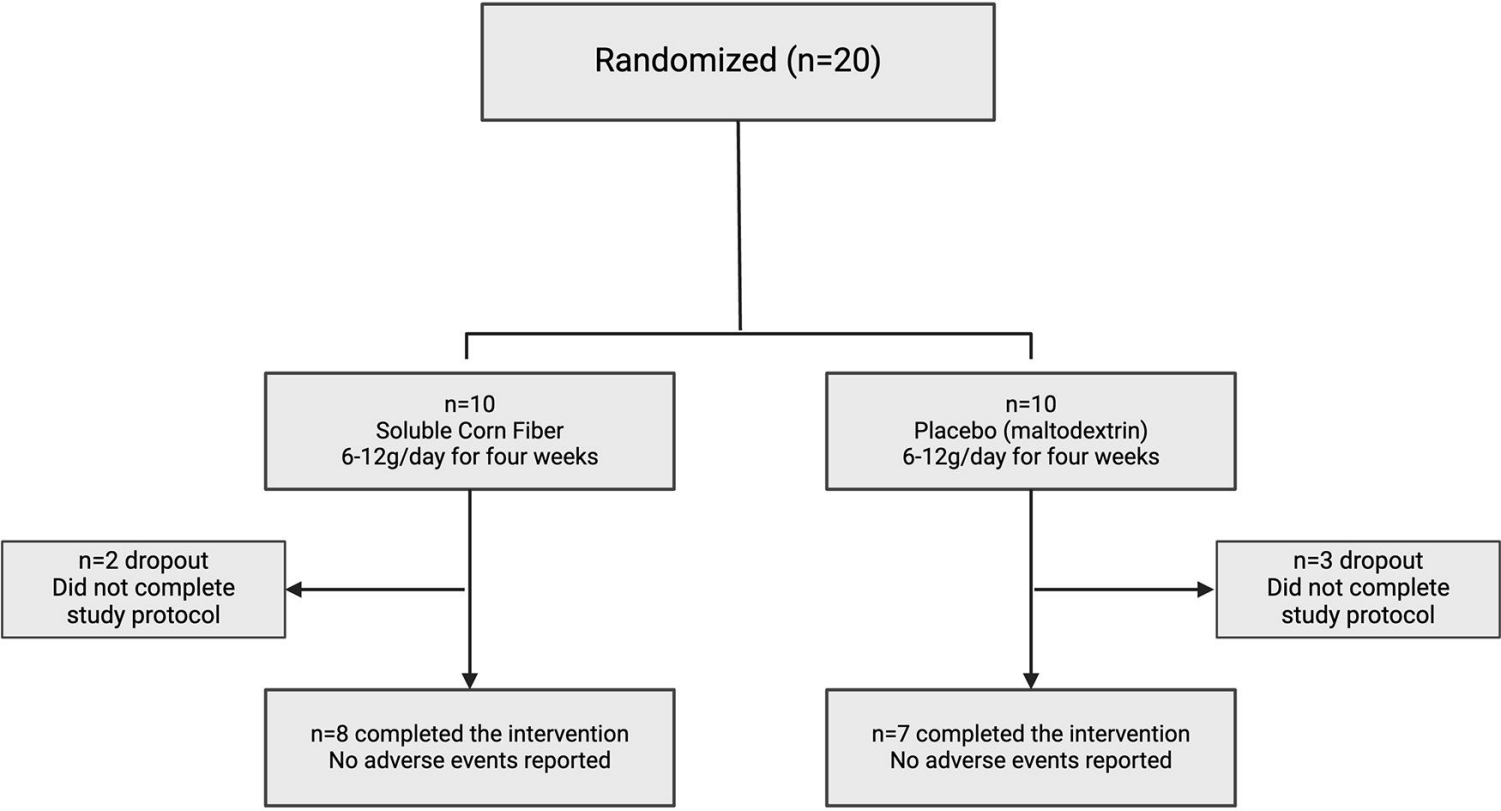
Flow chart of participant enrollment and retention. Twenty participants were enrolled and randomized 1:1 into the treatment (soluble corn fiber) or placebo (maltodextrin) arm. In the treatment arm, 2 participants dropped out of the intervention, and 8 participants completed the intervention. In the placebo arm, 3 participants dropped out of the intervention, and 7 participants completed the intervention. Figure created with biorender.com.

Twenty children were consented/assented to the study and were randomized, 10 to each study arm. Five children did not complete the 4–6 week intervention (75% retention, Figure 2). Dropouts were related to participants and their families voluntarily deciding not to complete the intervention for the full 4 weeks. Two participants dropped out in the fiber arm, three participants dropped out in the placebo arm (Figure 2). No adverse events were reported.

Participants consumed the fiber for a median of 34 days and a range of 28–42 days. The demographics and baseline values are summarized in Table 1. Adherence to the study protocol was calculated using packets returned (the number dispensed, returned used packets, and returned unused packets). Randomization allowed for reasonably balanced distribution of age and sex between the groups when considering the total enrolled male and female participants. In total, we enrolled 10 male and 5 female participants. In the fiber group, we had 6 males and 2 female participants. In the placebo arm, we had 4 male and 2 female participants. The average age was 11 y/o in both groups (range 7–13 in fiber, 7–15 in placebo; Student's *t*-test *p* = 0.727). Other key confounders were balanced across groups, including adherence to intervention (84% average compliance in fiber, 81% average in placebo; Student's *t*-test *p* = 0.727) and baseline fiber intake (13.7 g/day in fiber v. 11.15 g/day in placebo, Student's *t*-test *p* = 0.221). The mean adherence was 83% (median 85%) during the 4-week intervention (Table 1). Dietary fiber was calculated from the DSQ and the results are listed in Table 2. Overall, participants who completed the DSQ at each visit reported consuming fiber in their standard diet excluding the supplemented fiber, except for one participant. The DSQ does not discriminate across different sources or composition of fiber. For most participants who completed the DSQ at each visit, fiber intake modestly decreased at Visit 2 (average −1.08 g/day; range −5.65 to 2.25); only one participant reported increased dietary fiber at Visit 2 compared to Visit 1 (Table 2). This slight decrease was consistent across treatment arms (Student's *t*-test *p* = 0.647). We did not observe significant changes in ACQ scores between the placebo and fiber-supplemented group (*p* = 0.50, Student's *t*-test on the ΔACQ between treatment arms, Table 2).

**Table 1:** Participant demographics.

Participant ID	Status	Age	Sex	Race/Ethnicity	
1651	Completed	14	F	White	Hispanic/Latino
7b8f	Completed	13	M	Native American	
b561	Completed	15	F	White	Hispanic/Latino
7cb7	Did not complete	16	M	White	Hispanic/Latino
193e	Completed	13	F	White	
20e6	Did not complete	16	M	White	
3da0	Completed	11	M	Black	
20e1	Completed	11	M	White	Hispanic/Latino
03c5	Did not complete	16	M	White	
8570	Completed	11	M	Asian	
964c	Did not complete	11	M	Black	
c307	Completed	12	M	White	Hispanic/Latino
b244	Completed	10	M	White	
c791	Completed	10	M	White	Hispanic/Latino
c96d	Completed	7	M	White	
1704	Completed	13	F	White	
089e	Completed	7	M	Black	
e738	Completed	13	F	White	
5727	Completed	10	M	Black	
d44b	Did not complete	12	F	White	

**Table 2:** Individualized outcomes pre- and post-intervention.

Visit	Treatment	Acetic acid (μg/mL)	Δ Acetic acid (V2–V1)	Isovaleric acid (μg/mL)	Δ Isovaleric acid (V2–V1)	ACQ-6	Δ ACQ-6 (V2–V1)	Compliance	Compliance (bin)	Dietary fiber consumption	Δ Dietary fiber consumption (V2–V1)	Unifrac distance Visit 1–Visit 2
Visit1	Fiber	85.54	−2.10	0.18	−0.17	0.70	−0.40	93%	High	NA	NA	0.27
Visit2	Fiber	83.44		0.01		0.30				NA		
Visit1	Fiber	37.34	24.30	NA	NA	1.20	0.00	69%	Medium	13.01	−0.45	0.35
Visit2	Fiber	61.64		0.21		1.20				12.56		
Visit1	Fiber	47.77	5.60	0.26	−0.18	2.00	−0.80	86%	High	9.65	2.25	0.33
Visit2	Fiber	53.37		0.08		1.20				11.90		
Visit1	Fiber	46.98	28.22	0.22	−0.20	0.20	NA	96%	High	13.87	NA	0.32
Visit2	Fiber	75.20		0.02		NA				NA		
Visit1	Fiber	94.61	−35.32	0.22	−0.05	0.00	0.00	100%	High	22.25	−5.65	0.42
Visit2	Fiber	59.29		0.17		0.00				16.60		
Visit1	Fiber	72.82	18.68	0.42	−0.26	0.50	3.80	96%	High	22.69	−2.00	NA
Visit2	Fiber	91.50		0.16		4.30				20.69		
Visit1	Fiber	87.43	−7.71	0.07	0.11	0.50	−0.50	85%	High	14.30	−1.10	0.52
Visit2	Fiber	79.72		0.18		0.00				13.20		
Visit1	Fiber	NA	NA	NA	NA	1.20	0.00	50%	Low	NA	NA	0.41
Visit2	Fiber	NA		NA		1.20				NA		
Visit1	Placebo	49.12	18.26	0.10	0.01	0.80	−0.30	81%	Medium	11.70	NA	0.50
Visit2	Placebo	67.38		0.11		0.50				NA		
Visit1	Placebo	48.17	4.37	NA	NA	2.00	−0.80	64%	Low	10.78	−0.62	0.42
Visit2	Placebo	52.54		0.13		1.20				10.16		
Visit1	Placebo	110.46	16.20	0.09	0.05	0.00	0.00	92%	High	11.50	NA	NA
Visit2	Pacebo	126.66		0.14		0.00				NA		
Visit1	Placebo	40.29	53.79	0.16	0.32	0.50	2.20	71%	Medium	9.62	−0.76	0.51
Visit2	Placebo	94.08		0.48		2.70				8.86		
Visit1	Placebo	91.14	−3.32	0.05	0.03	0.80	−0.80	100%	High	NA	NA	0.35
Visit2	Placebo	87.82		0.08		0.00				NA		
Visit1	Placebo	65.32	13.78	0.35	−0.07	0.30	−0.30	100%	High	13.74	NA	0.34
Visit2	Placebo	79.10		0.28		0.00				NA		
Visit1	Placebo	39.96	NA	0.04	NA	2.00	−1.50	61%	Low	10.65	−0.32	NA
Visit2	Placebo	NA		NA		0.50				10.33		

### Individualized short chain fatty acid levels after a 4–6 week intervention with soluble corn fiber and placebo control

**Figure 3:**
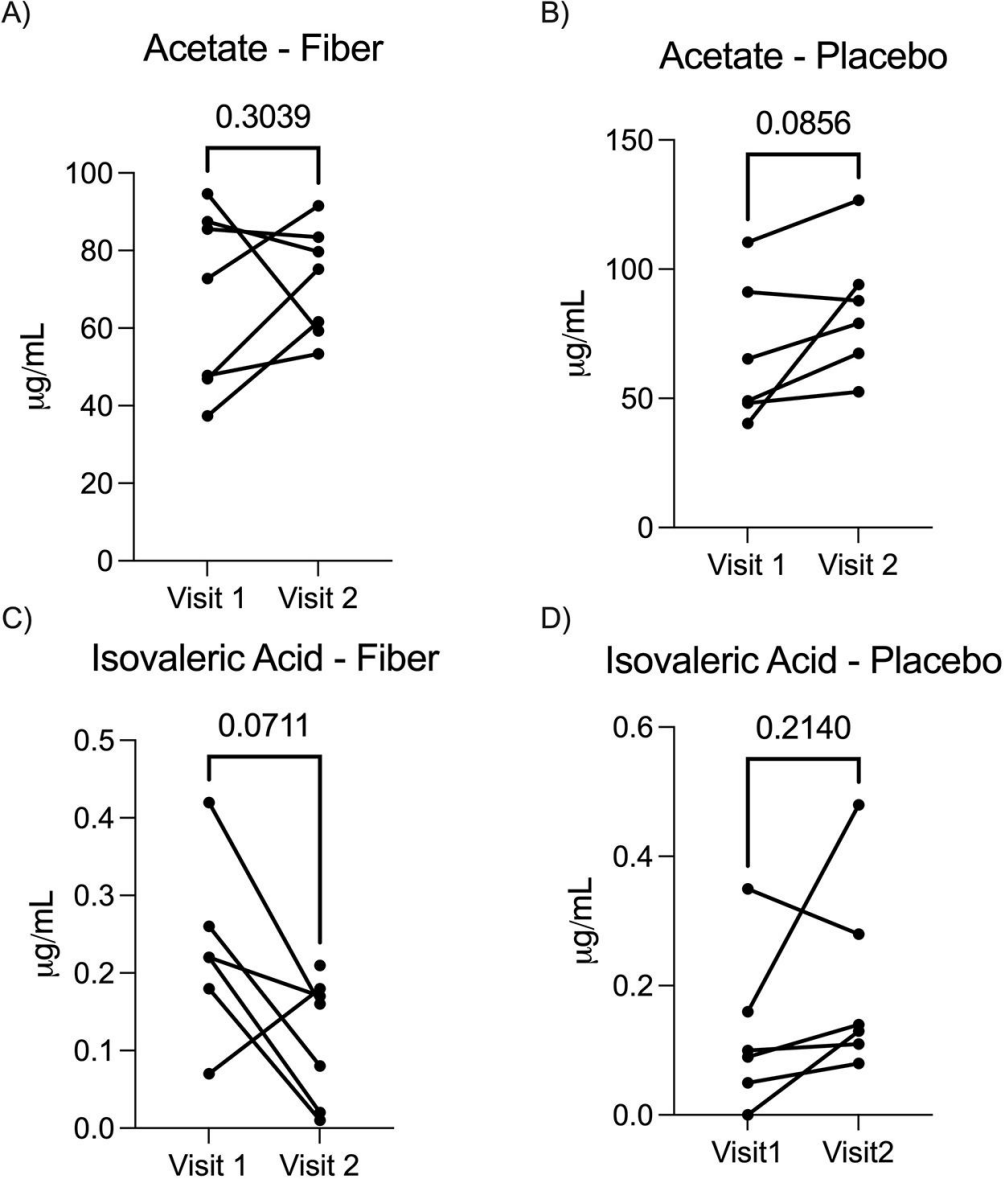
Pairwise analysis of serum acetate and isovaleric acid at visit 1 and visit 2 for each participant that completed a blood draw at both visits. In the SCF arm, *n* = 7 of 8 participants had blood drawn at both visits. In the placebo arm, *n* = 6 of 7 participants had blood drawn at both visits and are represented in this figure. **(A)** Changes in serum acetate in participants receiving soluble corn fiber were not significant at Visit 2 compared to Visit 1 (pairwise *t*-test *p* = 0.3039), **(B)** Changes in serum acetate in participants receiving placebo were not significant at Visit 2 compared to Visit 1 (pairwise *t*-test *p* = 0.0856), **(C)** Changes in serum isovaleric acid in participants receiving soluble corn fiber were not significant at Visit 2 compared to Visit 1 (pairwise *t*-test *p* = 0.0711). One participant had no detectable isovaleric acid at Visit 1. **(D)** Changes in serum isovaleric acid in participants receiving placebo were not significant at Visit 2 compared to Visit 1 (pairwise *t*-test *p* = 0.2140).

**Figure 4:**
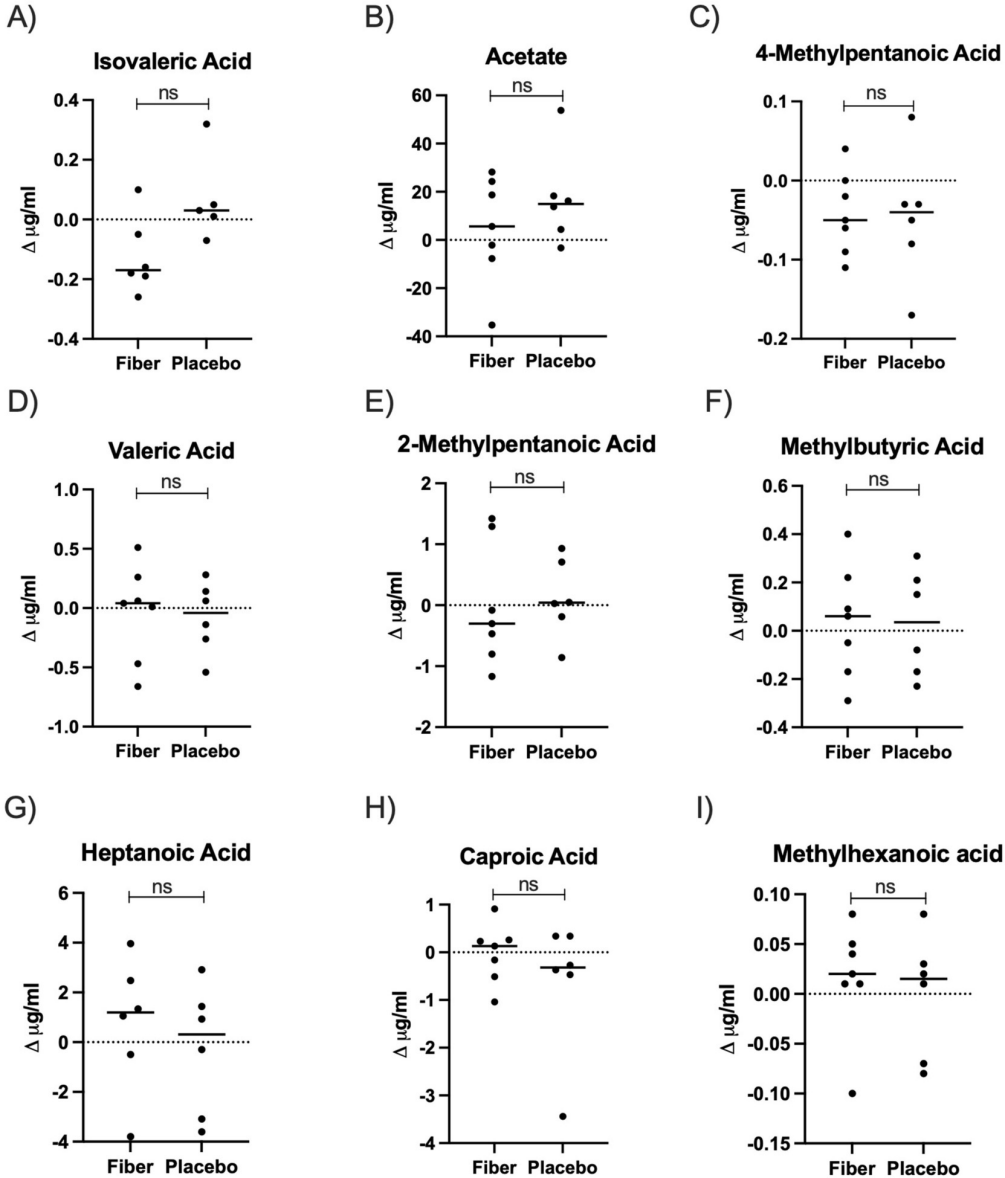
Serum SCFA analysis. Changes in all detectable serum SCFAs (Δ = Visit 2 – Visit 1) in participants receiving soluble corn fiber or placebo control. Not all SCFAs were detected across Visit 1 and Visit 2. When this occurred, a Δ could not be calculated. FDR corrected unpaired *t*-test *p* > 0.05 for all comparisons demonstrating no significant changes in serum SCFAs in participants receiving soluble corn fiber compared to placebo. **(A)** Isovaleric acid, **(B)** acetate, **(C)** 4-methylpentanoic acid, **(D)** valeric acid, **(E)** 2-methylpentanoic acid, **(F)** methylbutyric acid, **(G)** heptanoic acid, **(H)** caproic acid, and **(I)** methylhexanoic acid.

The primary outcome in our study was the quantification of circulating short chain fatty acids (SCFAs). We quantified fourteen SCFAs in serum collected at Visits 1 and 2. In the SCF arm, *n* = 7 of 8 participants had blood drawn at both visits. In the placebo arm, *n* = 6 of 7 participants had blood drawn at both visits. The SCFAs acetate, butyrate, and propionate have been most studied for their role in immune modulation in inflammatory disease, including asthma. Of these three, only acetate was detected in our assay. SCFAs can be produced through fermentation of protein products, and isovaleric acid is a primary product of protein fermentation. We observed high interpersonal variation in the quantity of acetate and isovaleric acid (Figure 3, Table 2). Of the 14 SCFAs measured, 9 were detected, but we did not observe significant changes at Visit 2 when compared to Visit 1 after correcting for multiple comparisons using the FDR method (unpaired *t*-test FDR *p* > 0.05 for all SCFAs, Figure 4). Not all SCFAs were detected across Visit 1 and Visit 2. When this occurred, a Δ could not be calculated.

### Individualized changes in the fecal microbiome after intervention with soluble corn fiber and placebo control

A secondary outcome of this study was changes in the gut microbiome diversity and composition following fiber treatment. Fecal samples were sequenced to an average of 82,207 sequences per sample (range 2,209–307,374 sequence/sample). Three samples did not have adequate sequence depth: one participant in the fiber arm was removed due to low sequence depth (<2,209) and one participant in the placebo arm was removed due to low sequence depth. Therefore, *n* = 7 (fiber) and *n* = 6 (placebo) are represented when comparing samples at Visit 2 (post-intervention). One additional participant in the placebo arm did not have a Visit 2 fecal sample. Therefore *n* = 7 (fiber) and *n* = 5 (placebo) are represented when a delta is calculated within an individual across Visits.

**Figure 5:**
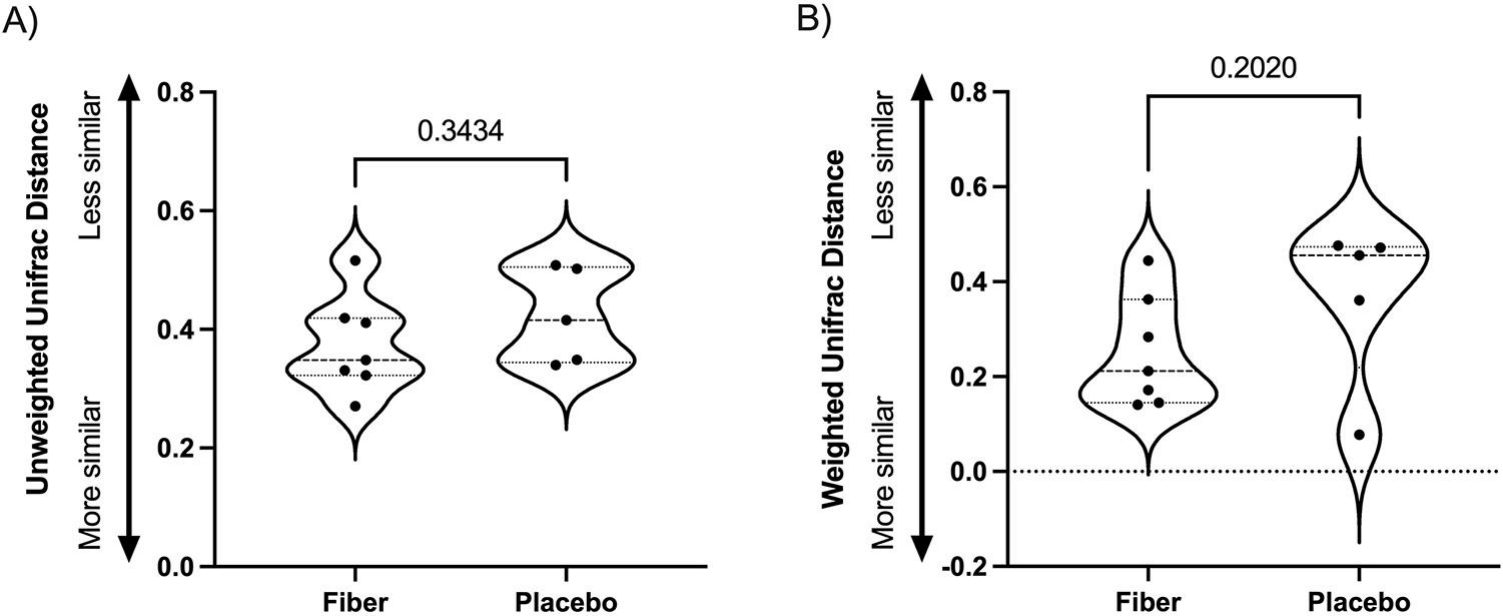
Distance of the gut microbiome at visit 2 compared to baseline. Unweighted **(A)** and Weighted **(B)** UniFrac distance at Visit 2 compared to baseline (Visit 1). One participant in the fiber arm was removed due to low sequence depth. In the placebo arm, one participant was removed due to low sequence depth, and one participant did not have a visit 2 fecal sample. Therefore, *n* = 7 (fiber) and *n* = 5 (placebo) are represented here. No significant differences in unweighted (Mann Whitney *p* = 0.343, Panel **A**) or weighted UniFrac distances were observed (Mann Whitney *p* = 0.202, Panel **B**).

**Figure 6:**
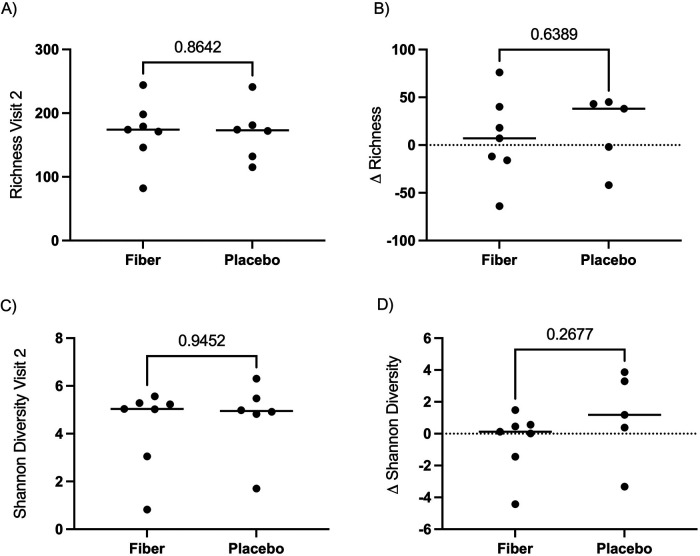
No significant change in alpha diversity from baseline across treatment arms. **(A)** We did not observe a significant change in richness at Visit 2 (post-intervention) across treatment arms (Mann Whitney *p* = 0.8462). **(B)** No significant change in richness was observed within individuals at Visit 2 compared to Visit 1 (Mann Whitney *p* = 0.6389) across treatment arms. **(C)** No significant change in Shannon diversity was observed at Visit 2 (post-intervention) across treatment arms (Mann Whitney *p* = 0.9452). **(D)** No significant change in Shannon Diversity was observed within individuals at Visit 2 compared to Visit 1 (Mann Whitney *p* = 0.2677) across treatment arms. One participant in the fiber arm was removed due to low sequence depth. In the placebo arm, one participant was removed due to low sequence depth, and one participant did not have a Visit 2 fecal sample. Therefore, *n* = 7 (fiber) and *n* = 6 (placebo) are represented in panels **A** and **C**, and *n* = 7 (fiber) and *n* = 5 (placebo) are represented in panels **B** and **D**.

To determine whether the gut microbiome composition changed significantly between Visit 1 and Visit 2 in the participants receiving SCF or placebo, we calculated Weighted and Unweighted UniFrac distances between each pair of samples. We did not observe a significant change in the gut microbiome of participants who consumed dietary fiber compared to the placebo control (Figure 5A,B and Supplementary Figure S1, Wilcoxon Rank Sum test of Δ Weighted UniFrac *p* = 0.202, Δ Unweighted UniFrac *p* = 0.343; PERMANOVA of Weighted UniFrac *p* = 0.310, PERMANOVA of Unweighted UniFrac *p* = 0.601 at Visit 2). Next, we asked whether alpha (within-sample) diversity changed between Visit 1 and Visit 2 in participants receiving SCF or placebo. Richness (the number of unique ASVs) and Shannon diversity (which takes into account the richness and distribution of ASVs) were used to measure alpha diversity, and we did not observe significant differences at Visit 2 between individuals consuming placebo compared to fiber (Figure 6A Mann Whitney *p* = 0.8642, Figure 6C Mann Whitney *p* = 0.9452). We also compared the change in alpha diversity within individuals (Δ = Visit 2-Visit 1). Again, we did not observe a significant difference in the alpha diversity delta across treatment arms (Figure 6B Mann Whitney *p* = 0.6389, Figure 6C Mann Whitney *p* = 0.2377).

**Figure 7:**
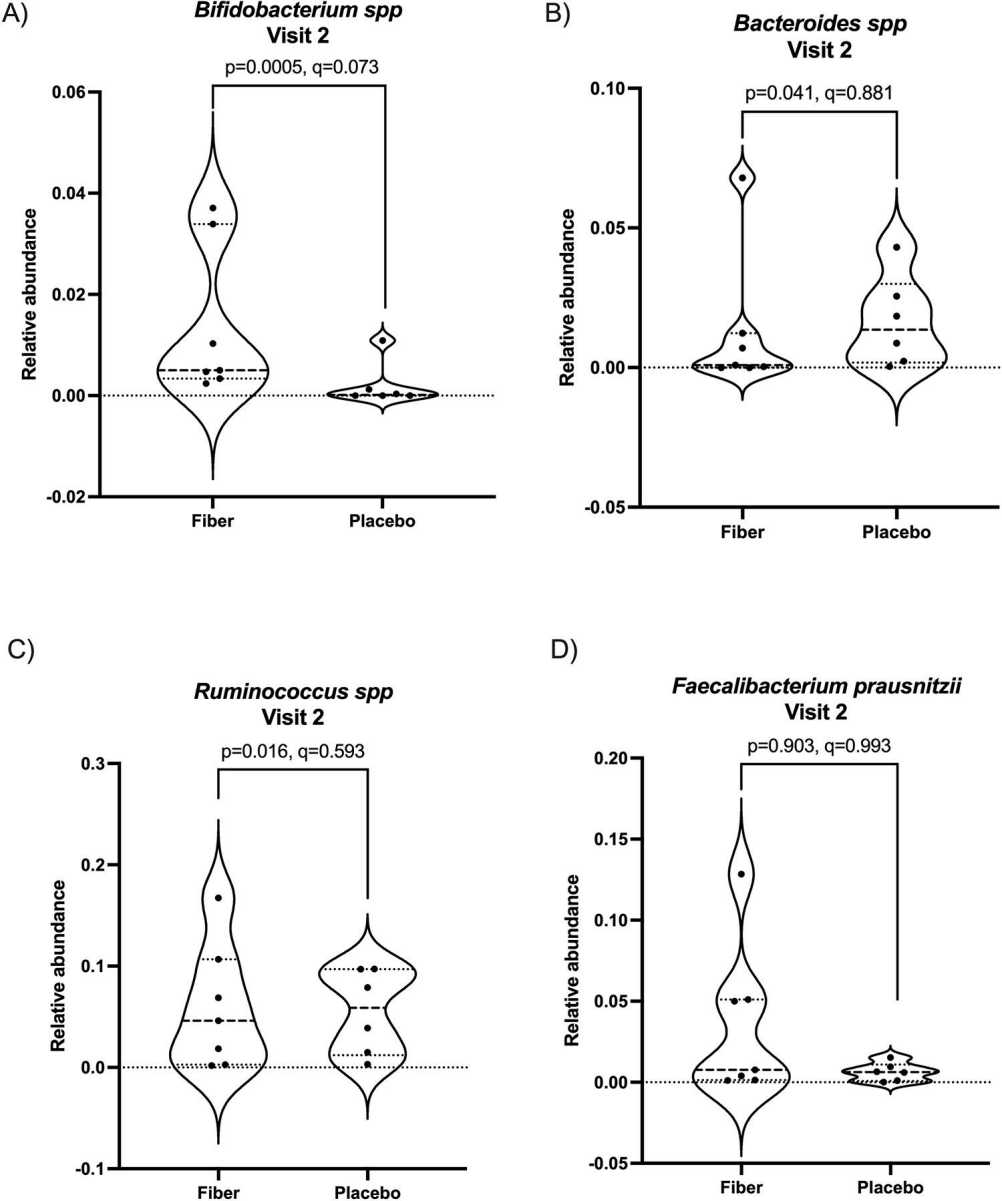
Differential abundance of key gut microbial taxa after four-week intervention with dietary fiber. ANCOMBC was used to determine differential abundance of taxa collapsed at L7 (approximately species level) after fiber intervention. FDR was applied to correct for multiple comparisons. **(A)** Bifidobacterium spp. were trending toward significantly enriched in the gut microbiome of participants consuming dietary fiber (*p* = 0.0005, *q* = 0.073), **(B)** *Bacteroides spp*. were not significantly different between the two groups (*p* = 0.041, *q* = 0.881), **(C)** Ruminococcus spp were not significantly different between the two groups (*p* = 0.016, *q* = 0.593), and **(D)** *Faecalibacterium prausnitzii* were not significantly different between the two groups (p = 0.903, q = 0.993).

Finally, we were interested in whether specific microbial taxa were differentially abundant in the gut microbiome of participants receiving SCF or placebo. We used ANCOMBC to compare the gut microbiome composition collapsed to the level 7 (which approximates species level taxonomy) at Visit 2 between participants receiving SCF and placebo. A total of 150 taxa were detected (Supplementary File S1). *Bifidobacterium* spp. were elevated in participants receiving SCF compared to placebo, but this did not reach statistical significance after correcting for multiple comparisons (*p* = 0.0004, FDR *q* = 0.073, ANCOMBC). In Figure 7, we highlight differences in the relative abundance of four key gut microbial taxa (*Bifidobacterium*, *Bacteroides*, *Ruminococcus*, and *Faecalibacterium prausnitzii*) commonly involved in GI health. When comparing taxa collapsed to level 7 at Visit 1 using ANCOMBC, we did not observe any statistical significance after correcting for multiple comparisons (FDR *q* ≥ 0.987 for all features). Focusing on *Bifidobacterium* spp., we did not observe statistically significant differences in this feature at Visit 1 between SCF and placebo (ANCOMBC *p* = 0.07, FDR *q* = 0.990). We compared the relative abundance of *Bifidobacterium* of our paired fecal samples from individuals at Visit 1 compared to Visit 2 who consumed SCF or placebo. We did not see a statistically significant increase in either group at the individual level (Wilcoxon matched pairs signed rank test; SCF *p* = 0.371, placebo *p* = 0.250).

### Soluble corn fiber did not alter the nasal microbiome

We next assessed nasal microbiome composition and diversity. Five of 8 participants in the Fiber arm and 6 of 7 participants in the Placebo arm had at least a Visit 1 specimen. Three participants in the fiber arm and three participants in the placebo arm had paired samples at Visit 1 and Visit 2. We calculated weighted and unweighted UniFrac distances and assessed compositional differences at Visit 2 between individuals receiving fiber. We did not observe significant differences using weighted (PERMANOVA *p* = 0.310, pseudo-*F* = 1.394) or unweighted Unifrac (PERMANOVA *p* = 0.601, pseudo-*F* = 0.759). Similarly, we did not observe differences in alpha diversity metrics at Visit 2 between individuals receiving fiber or placebo (Richness, Kruskal Wallis *p* = 0.827; Shannon diversity, Kruskal Wallis *p* = 0.127). Because only *n* = 3 participants in each treatment arm had a Visit 2 sample sequenced, we did not perform ANCOMBC to test for differential abundance. The taxonomic bar plots tend to show similar dominant taxa at Visit 1 and Visit 2 that are unique to the participant (Supplementary Figure S2). For example, participant 3da0 had a nasal microbiome dominated by *Moraxella* (tan) at Visit 1 that was unchanged in Visit 2. Other participants had nasal microbiota dominated by *Corynebacterium* (green) or *Staphylococcus* (purple) that remained relatively stable across visits regardless of treatment arm. We generated a PCoA of Unweighted Unifrac distances and did not observe clustering by treatment arm at Visit 1 or Visit 2 (Supplementary Figure S3).

## Discussion

This pilot randomized controlled trial investigated the effects of SCF supplementation on the gut microbiome, serum SCFAs, nasal microbiome, and clinical asthma control in children with asthma. Despite the known benefits of fiber on gut and systemic health, our study did not find significant differences between the intervention and placebo groups in any of the primary or secondary outcomes. These findings contribute to our understanding of the complexity of dietary interventions and their impact on chronic inflammatory diseases such as asthma and highlight potentially important limitations that will inform future clinical studies on modulating the gut microbiome-lung axis by supplementing the normal diet with fiber. Our modest findings were somewhat unexpected given the dominant effect of dietary patterns on gut microbiota composition and function (48–51). Though, we did observe a trending increase in *Bifidobacterium* in participants receiving the SCF supplement compared to controls. This finding aligns with several studies and a recent meta-analysis, which demonstrated an overall trend of increased *Bifidobacterium* and other taxa across multiple studies of fiber supplementation (23–25, 52).

Two key observations that may have limited our study are (1) the potential insufficiency of the dose of fiber used, which was a supplement of approximately half of recommended dietary daily intake, and (2) supplementation with a single type of fiber (soluble corn fiber), rather than a mixture. The fiber dose we used may be insufficient to influence the gut microbiome and therefore generate adequate levels of SCFAs to impact airway inflammation. In addition, habitual fiber intake moderately decreased in many of the participants when measured after the intervention, though this did not reach statistical significance. The moderate reduction in fiber intake during the intervention period, along with the conservative dosage we used, could have offset the benefit of the fiber supplementation. Further, our dosing strategy didn't guarantee a participant would reach 100% of the daily recommended intake. For example, if a participant at baseline consumed 25% of the recommended fiber per day and continued at this level (or lower) during the study, they would have consumed a maximum of 75% recommended daily fiber if in the intervention arm or 25% if in the placebo arm. Dose-dependent effects of dietary fiber have been documented on both SCFA levels and changes in the gut microbiome (37, 53). In one study, supplementing with higher doses of soluble corn fiber (0, 10, 20 g) increased the relative abundance of *Parabacteroides* in the gut microbiome of healthy pubertal females (54). Another study in healthy adult participants demonstrated that different doses of fiber (0, 10, 20, 35, 50 g/day) differentially alter the gut microbiome composition and type of SCFA produced. They found that, of the four dietary fibers used, the dominant effect plateaued at 35 g/day (53). This study also highlighted the importance of dietary fiber structure in modifying the gut microbiome. Crystalline maize resulted in enrichment of *Eubacterium* whereas crosslinked tapioca starch resulted in enrichment of *Parabacteroides* (53). A crossover study of 18 healthy participants given arabinoxylan, followed by long-chain inulin, then a third intervention with a diverse mix of fibers demonstrated differential microbial, metabolic, and immune responses across the three treatment arms (5), Arabinoxylan consumption lowered overall low density lipoproteins (LDL) and increased average relative abundance of *Bacteroides xylanisolvens* and *Roseburia* in a dose-dependent manner. Long chain inulin resulted in increased *Bifidobacterium pseudocatenulatum*, *B. bifidum*, and the butyrate-producing *Clostridium* cluster XIVa. Although it could be rationalized that mixed fiber supplements could be more effective in regulating the gut microbial communities and production of more diverse SCFAs, in this study, the mixed fiber supplement had a more modest effect on the gut microbiome and host immune response that mirrored some of the changes of each purified fiber (5). These observations highlight the need for a precision approach in fiber dosage and structure in future trials.

The high interindividual variability in gut microbiome responses to fiber supplementation is noteworthy. Other studies of fiber supplementation, rather than whole dietary intervention, demonstrate that several independent factors, such as lifestyle and nutrition, can influence the response of the gut microbiome and inflammatory outcomes (26, 55–57). A study in genetically identical gnotobiotic mice harboring two distinct gut microbial communities found that interindividual variation in the gut microbiome is linked to differential effects of fiber on host metabolic phenotypes (58). Studies in humans also show variable responses and effect sizes, but are confounded by different fibers, dosages, and duration of intervention (53, 54, 59). This effect was recently summarized in a meta-analysis of 21 fiber interventions from 12 human studies (55). Given our small sample size, we were unable to assess whether interindividual variation of the gut microbiome influenced the variable responses that we observed; however, future studies with larger fiber supplementation doses in larger cohorts will allow us to consider the baseline microbiome in our outcome variables. Likely, personalized nutrition approaches, which consider individual baseline microbiome compositions and fiber consumption, will be necessary to achieve consistent and clinically meaningful outcomes.

The timing of blood sample collection relative to the last fiber intake is another important consideration. We expected to observe an increase in serum SCFA that reflected changes occurring in the gut microbiome. Given that we did not observe significant changes in the gut microbiome, it is not surprising that we did not observe changes in serum SCFA. Serum collection times were not standardized in this study to the last dose of fiber (or placebo) ingested and we did not record when participants last consumed a fiber supplement, when they consumed fiber in their normal diet, or consumed their last meal. This likely contributed to the observed variability in the serum SCFA results. Future studies will standardize the timing of the last fiber supplement to the second blood draw and we will collect a 24 h dietary recall at the time of each blood draw. This could be an important limitation to our SCFA measurements, because studies have shown that gut microbiome and SCFA-driven improvements are not observed until after 24 h, perhaps because of transit time to the lower intestine for microbial fermentation (60, 61). Further, the majority of SCFAs produced in the colon are metabolized by gut epithelial cells, and our conservative fiber dose may not have resulted in SCFA produced in excess to be detected within our detection limits (62). Studies report SCFAs in the serum at the lower end of µg/mL after fiber intake. Paired with a conservative fiber dose, this could have resulted in non-detection of propionate and butyrate. Future studies should standardize sample collection times to fiber and meal intake to more accurately measure the acute effects of fiber intake on SCFA levels.

Our findings also suggest that without significant changes in the gut microbiome, downstream effects on airway inflammation and asthma control are unlikely. Although this study was not designed to detect differences in clinical asthma outcomes (ACQ), we included this as a secondary outcome regardless. Children with stable asthma who were not planning to change their asthma medicines in the next 4–6 weeks were enrolled. This allowed for isolating the impact of the fiber intervention but also made achieving improvement in asthma control compared to excellent baseline asthma control more difficult. Starting with participants with well controlled asthma (ACQ < 1.5) likely made it more difficult to detect a meaningful improvement in ACQ scores (which would be a decrease in a score by 0.5 minimally). ACQ is only one clinical measure to assess asthma control or inflammation. Future studies will include FEV1% to assess pulmonary function and more robust immunological analyses. Nasal cytokine analysis may give an approximation of pulmonary inflammation, as hypothesized by the “unified airway hypothesis”, which proposes that upper and lower airway disease reflect a single pathological process (63–65). The mechanism of SCFA-driven reduction in inflammation is likely through G protein coupled receptor (GPCR) signaling or perhaps through epigenetic modification [HDAC inhibition; (11, 18)]. Thus, it would be important to assess these pathways in circulating and airway immune cells. Given the complex nature of asthma, which involves multiple inflammatory pathways and triggers, it is possible that a multi-faceted approach, inclusion of additional outcome measures, and combining dietary interventions with other treatments, may be necessary to achieve meaningful clinical improvements.

There were several limitations to note for this pilot trial. First, the small sample size limits conclusions, and we were not powered to perform *ad hoc* analyses on subgroups of participants, such as responders and non-responders. Second, while we collected unused fiber/placebo sachets to estimate adherence to the fiber intake (∼80%); this could underestimate actual adherence. Participants could have discarded powder without consumption. Future studies should include objective measures of adherence, such as inclusion of an e-diary with timestamped photos when packets were consumed. Third, the timing of the phlebotomy relative to the last fiber intake timing could miss a transient increase in serum SCFA. Fecal samples were not collected or stored for metabolomic analysis, so we could not measure fecal levels of SCFAs. Measuring fecal SCFAs could also be important as they modify the GI immune landscape by enhancing production of Tregs (66). These changes have been shown to contribute to systemic immune responses, for example, by enhancing protection against peanut allergy as an adjuvant to oral immunotherapy (66). Despite these limitations, there remains strong epidemiologic evidence and mechanistic data to suggest that the low-fiber industrialized diet may have an important role in initiating and maintaining the airway inflammation seen in asthma (10, 11, 16, 17, 67). The findings from this study suggest that a different fiber source and/or higher fiber dose may be needed in children with asthma to influence the gut microbiome-airway inflammation axis. Despite these limitations, our study did have a few notable strengths. This study was completed using a patient-administered intervention providing real-world applicability for intervening with dietary fiber interventions in pediatric populations. Mean compliance was also good among the SCF arm suggesting that the fiber is tolerable and easy to incorporate into daily dietary practices as drink mixes. Lastly, the implementation of this trial in a clinical setting shows the feasibility of integrating dietary fiber intake guidance in a setting where high-risk pediatric patients may receive other systemic health benefits from SCF or similar fermentable prebiotic fibers.

## Conclusion

A pilot randomized trial of supplementation of dietary fiber using 50% of daily intake with soluble corn fiber did not demonstrate significant changes to the gut microbiome or any of the anticipated downstream effects such as serum SCFA levels, nasal microbiome, or clinical asthma outcomes. Nonetheless, the results from this trial provide valuable insight for future research. Higher fiber doses, fiber supplementation types, consideration of baseline microbiome composition, consideration of baseline fiber intake in individuals, and larger sample sizes will be critical in subsequent studies.

## Data Availability

The datasets presented in this study can be found in online repositories. The names of the repository/repositories and accession number(s) can be found below: https://www.ncbi.nlm.nih.gov/, PRJNA1258290.

## Supplementary material

Supplementary File S1 ANCOMBC results for fecal microbiome at Visit 2.

Supplementary Figure S1 No clustering in fecal samples by treatment arm at Visit 1 or Visit 2. (A) PCoA of Unweighted UniFrac of fecal samples at Visit 1 do not show clear patterns of separation between treatment arms. (B) PCoA of Unweighted UniFrac of fecal samples at Visit 2 do not show clear patterns of separation between treatment arms.

Supplementary Figure S2 Taxonomic bar plot of the nasal microbiome in participants receiving fiber supplement or placebo control. Three participants in the fiber arm and three participants in the placebo arm had paired samples at Visit 1 and Visit 2. Five of 8 participants in the Fiber arm and 6 of 7 participants in the Placebo arm had at least a Visit 1 specimen. In the paired samples, the nasal microbiome did not substantially change after the intervention period nor did the intervention type influence the nasal microbiome composition.

Supplementary Figure S3 No clustering in nasal samples by treatment arm at Visit 1 or Visit 2. (A) PCoA of Unweighted UniFrac of nasal samples at Visit 1 do not show clear patterns of separation between treatment arms. (B) PCoA of Unweighted UniFrac of nasal samples at Visit 2 do not show clear patterns of separation between treatment arms.

## References

[1] Most recent national asthma data. (2024). Available online at: https://www.cdc.gov/asthma/most_recent_national_asthma_data.htm (Accessed January 07, 2026).

[2] Johnson CC Havstad SL Ownby DR Joseph CLM Sitarik AR Biagini Myers J Pediatric asthma incidence rates in the United States from 1980 to 2017. J Allergy Clin Immunol. (2021) 148(5):1270–80. 10.1016/j.jaci.2021.04.027 33964299 PMC8631308

[3] Pate CA Zahran HS Qin X Johnson C Hummelman E Malilay J. Asthma surveillance - United States, 2006-2018. Morb Mortal Wkly Rep Surveill Summ. (2021) 70(5):1–32. 10.15585/mmwr.ss7005a1 PMC8480992 34529643

[4] Asthma. (2021). Available online at: https://www.cdc.gov/asthma/nhis/default.htm (Accessed January 07, 2026).

[5] Lancaster SM Lee-McMullen B Abbott CW Quijada JV Hornburg D Park H Global, distinctive, and personal changes in molecular and microbial profiles by specific fibers in humans. Cell Host Microbe. (2022) 30(6):848–62.e7. 10.1016/j.chom.2022.03.036 35483363 PMC9187607

[6] Bustamante-Sanchez A Villegas-Mora BE Martínez-Guardado I Tornero-Aguilera JF Ardigò LP Nobari H Physical activity and nutritional pattern related to maturation and development. Sustainability. (2022) 14(24):16958. 10.3390/su142416958

[7] González Olmo BM Butler MJ Barrientos RM. Evolution of the human diet and its impact on gut microbiota, immune responses, and brain health. Nutrients. (2021) 13(1):196. 10.3390/nu13010196 33435203 PMC7826636

[8] Li H Tang X Guo X Zhang M Zhang M Nie J Association of dietary patterns with chronic respiratory health among U.S. adults. Front Immunol. (2024) 15:1457860. 10.3389/fimmu.2024.1457860 39712005 PMC11659122

[9] Furusawa Y Obata Y Fukuda S Endo TA Nakato G Takahashi D Commensal microbe-derived butyrate induces the differentiation of colonic regulatory T cells. Nature. (2013) 504(7480):446–50. 10.1038/nature12721 24226770

[10] Trompette A Gollwitzer ES Pattaroni C Lopez-Mejia IC Riva E Pernot J Dietary fiber confers protection against flu by shaping Ly6c− patrolling monocyte hematopoiesis and CD8+ T cell metabolism. Immunity. (2018) 48(5):992–1005.e8. 10.1016/j.immuni.2018.04.022 29768180

[11] Trompette A Gollwitzer ES Yadava K Sichelstiel AK Sprenger N Ngom-Bru C Gut microbiota metabolism of dietary fiber influences allergic airway disease and hematopoiesis. Nat Med. (2014) 20(2):159–66. 10.1038/nm.3444 24390308

[12] Spencer CN McQuade JL Gopalakrishnan V McCulloch JA Vetizou M Cogdill AP Dietary fiber and probiotics influence the gut microbiome and melanoma immunotherapy response. Science. (2021) 374(6575):1632–40. 10.1126/science.aaz7015 34941392 PMC8970537

[13] Makki K Deehan EC Walter J Bäckhed F. The impact of dietary fiber on gut Microbiota in host health and disease. Cell Host Microbe. (2018) 23(6):705–15. 10.1016/j.chom.2018.05.012 29902436

[14] Hosseini-Esfahani F Koochakpoor G Daneshpour MS Mirmiran P Sedaghati-Khayat B Azizi F. The interaction of fat mass and obesity associated gene polymorphisms and dietary fiber intake in relation to obesity phenotypes. Sci Rep. (2017) 7(1):18057. 10.1038/s41598-017-18386-8 29273742 PMC5741758

[15] Carlson JL Erickson JM Lloyd BB Slavin JL. Health effects and sources of prebiotic dietary fiber. Curr Dev Nutr. (2018) 2(3):nzy005. 10.1093/cdn/nzy005 30019028 PMC6041804

[16] Schenzel A Geiger A Nendel E Yang Z Krammer S Leberle A Fiber rich food suppressed airway inflammation, GATA3+Th2 cells, and Fc*ε*RI*α*+ eosinophils in asthma. Front Nutr. (2024) 11:1367864. 10.3389/fnut.2024.1367864 38757128 PMC11097976

[17] Wen S Yuan G Li C Xiong Y Zhong X Li X. High cellulose dietary intake relieves asthma inflammation through the intestinal microbiome in a mouse model. PLoS One. (2022) 17(3):e0263762. 10.1371/journal.pone.0263762 35271579 PMC8912215

[18] McKenzie C Tan J Macia L Mackay CR. The nutrition-gut microbiome-physiology axis and allergic diseases. Immunol Rev. (2017) 278(1):277–95. 10.1111/imr.12556 28658542

[19] Fong FLY Kirjavainen PV El-Nezami H. Immunomodulation of Lactobacillus rhamnosus GG (LGG)-derived soluble factors on antigen-presenting cells of healthy blood donors. Sci Rep. (2016) 6(1):1–8. 10.1038/srep22845 26961406 PMC4785377

[20] Ludwig IS Broere F Manurung S Lambers TT van der Zee R van Eden W. Lactobacillus rhamnosus GG-derived soluble mediators modulate adaptive immune cells. Front Immunol. (2018) 9:361211. 10.3389/fimmu.2018.01546 PMC6048560 30042761

[21] Sun S Luo L Liang W Yin Q Guo J Rush AM Bifidobacterium alters the gut microbiota and modulates the functional metabolism of T regulatory cells in the context of immune checkpoint blockade. Proc Natl Acad Sci U S A. (2020) 117(44):27509–15. 10.1073/pnas.1921223117 33077598 PMC7959554

[22] Fanning S Hall LJ van Sinderen D. Bifidobacterium breve UCC2003 surface exopolysaccharide production is a beneficial trait mediating commensal-host interaction through immune modulation and pathogen protection. Gut Microbes. (2012) 3(5):420–5. 10.4161/gmic.20630 22713271

[23] So D Whelan K Rossi M Morrison M Holtmann G Kelly JT Dietary fiber intervention on gut microbiota composition in healthy adults: a systematic review and meta-analysis. Am J Clin Nutr. (2018) 107(6):965–83. 10.1093/ajcn/nqy041 29757343

[24] Bouhnik Y Flourié B Riottot M Bisetti N Gailing M Guibert A Effects of fructo-oligosaccharides ingestion on fecal bifidobacteria and selected metabolic indexes of colon carcinogenesis in healthy humans. Nutr Cancer. (1996) 26(1):21–9. 10.1080/01635589609514459 8844718

[25] Walton GE van den Heuvel EG Kosters MH Rastall RA Tuohy KM Gibson GR. A randomised crossover study investigating the effects of galacto-oligosaccharides on the faecal microbiota in men and women over 50 years of age. Br J Nutr. (2012) 107(10):1466–75. 10.1017/S0007114511004697 21910949

[26] Oliver A Chase AB Weihe C Orchanian SB Riedel SF Hendrickson CL High-fiber, whole-food dietary intervention alters the human gut microbiome but not fecal short-chain fatty acids. mSystems. (2021) 6(2). 10.1128/mSystems.00115-21 PMC8546969 33727392

[27] Cabana MD McKean M Caughey AB Fong L Lynch S Wong A Early probiotic supplementation for eczema and asthma prevention: a randomized controlled trial. Pediatrics. (2017) 140(3):e20163000. 10.1542/peds.2016-3000 28784701 PMC5574725

[28] Drago L Cioffi L Giuliano M Pane M Amoruso A Schiavetti I The probiotics in pediatric asthma management (PROPAM) study in the primary care setting: a randomized, controlled, double-blind trial with LS01 (DSM 22775) and B632 (DSM 24706). J Immunol Res. (2022) 2022:3837418. 10.1155/2022/3837418 35083341 PMC8786459

[29] Durack J Kimes NE Lin DL Rauch M McKean M McCauley K Delayed gut microbiota development in high-risk for asthma infants is temporarily modifiable by Lactobacillus supplementation. Nat Commun. (2018) 9(1):1–9. 10.1038/s41467-018-03157-4 29453431 PMC5816017

[30] Liu A Ma T Xu N Jin H Zhao F Kwok L-Y Adjunctive probiotics alleviates asthmatic symptoms via modulating the gut microbiome and serum metabolome. Microbiol Spectr. (2021) 9:e00859-21. 10.1128/Spectrum.00859-21 34612663 PMC8510161

[31] Smith PM Howitt MR Panikov N Michaud M Gallini CA Bohlooly-Y M The microbial metabolites, short-chain fatty acids, regulate colonic treg cell homeostasis. Science. (2013) 341(6145):569–73. 10.1126/science.1241165 23828891 PMC3807819

[32] Halnes I Baines KJ Berthon BS MacDonald-Wicks LK Gibson PG Wood LG. Soluble fibre meal challenge reduces airway inflammation and expression of GPR43 and GPR41 in asthma. Nutrients. (2017) 9(1):57. 10.3390/nu9010057 28075383 PMC5295101

[33] Thorburn AN McKenzie CI Shen S Stanley D Macia L Mason LJ Evidence that asthma is a developmental origin disease influenced by maternal diet and bacterial metabolites. Nat Commun. (2015) 6(1):7320. 10.1038/ncomms8320 26102221

[34] van de Pol MA Lutter R Smids BS Weersink EJ van der Zee JS. Synbiotics reduce allergen-induced T-helper 2 response and improve peak expiratory flow in allergic asthmatics. Allergy. (2011) 66(1):39–47. 10.1111/j.1398-9995.2010.02454.x 20716319

[35] McLoughlin R Berthon BS Rogers GB Baines KJ Leong LEX Gibson PG Soluble fibre supplementation with and without a probiotic in adults with asthma: a 7-day randomised, double blind, three way cross-over trial. EBioMedicine. (2019) 46:473–85. 10.1016/j.ebiom.2019.07.048 31375426 PMC6712277

[36] Berthon BS McLoughlin RF Jensen ME Hosseini B Williams EJ Baines KJ The effects of increasing fruit and vegetable intake in children with asthma: a randomized controlled trial. Clin Exp Allergy. (2021) 51(9):1144–56. 10.1111/cea.13979 34197676

[37] Juniper EF O’Byrne PM Guyatt GH Ferrie PJ King DR. Development and validation of a questionnaire to measure asthma control. Eur Respir J. (1999) 14(4):902–7. 10.1034/j.1399-3003.1999.14d29.x 10573240

[38] Thompson FE Midthune D Kahle L Dodd KW. Development and evaluation of the national cancer institute’s dietary screener questionnaire scoring algorithms. J Nutr. (2017) 147(6):1226–33. 10.3945/jn.116.246058 28490673 PMC5443466

[39] Thompson LR Sanders JG McDonald D Amir A Ladau J Locey KJ A communal catalogue reveals Earth’s multiscale microbial diversity. Nature. (2017) 551:457–63. 10.1038/nature24621 29088705 PMC6192678

[40] Gu H Jasbi P Patterson J Jin Y. Enhanced detection of short-chain fatty acids using gas chromatography mass spectrometry. Curr Protoc. (2021) 1(6):e177. 10.1002/cpz1.177 34165916 PMC8238372

[41] Bolyen E Rideout JR Dillon MR Bokulich NA Abnet CC Al-Ghalith GA Reproducible, interactive, scalable and extensible microbiome data science using QIIME 2. Nat Biotechnol. (2019) 37(8):852–7. 10.1038/s41587-019-0209-9 31341288 PMC7015180

[42] Callahan BJ McMurdie PJ Rosen MJ Han AW Johnson AJ Holmes SP. DADA2: high-resolution sample inference from illumina amplicon data. Nat Methods. (2016) 13(7):581–3. 10.1038/nmeth.3869 27214047 PMC4927377

[43] Janssen S McDonald D Gonzalez A Navas-Molina JA Jiang L Xu ZZ Phylogenetic placement of exact amplicon sequences improves associations with clinical information. mSystems. (2018) 3(3):2047–217X–2–16. 10.1128/mSystems.00021-18 PMC5904434 29719869

[44] DeSantis TZ Hugenholtz P Larsen N Rojas M Brodie EL Keller K Greengenes, a chimera-checked 16S rRNA gene database and workbench compatible with ARB. Appl Environ Microbiol. (2006) 72(7):5069–72. 10.1128/AEM.03006-05 16820507 PMC1489311

[45] Lozupone C Lladser ME Knights D Stombaugh J Knight R. Unifrac: an effective distance metric for microbial community comparison. ISME J. (2011) 5(2):169–72. 10.1038/ismej.2010.133 20827291 PMC3105689

[46] Vázquez-Baeza Y Pirrung M Gonzalez A Knight R. EMPeror: a tool for visualizing high-throughput microbial community data. Gigascience. (2013) 2(1):16. 10.1186/2047-217X-2-16 24280061 PMC4076506

[47] Lin H Peddada SD. Analysis of compositions of microbiomes with bias correction. Nat Commun. (2020) 11(1):3514. 10.1038/s41467-020-17041-7 32665548 PMC7360769

[48] David LA Maurice CF Carmody RN Gootenberg DB Button JE Wolfe BE Diet rapidly and reproducibly alters the human gut microbiome. Nature. (2014) 505(7484):559–63. 10.1038/nature12820 24336217 PMC3957428

[49] Wang DD Nguyen LH Li Y Yan Y Ma W Rinott E The gut microbiome modulates the protective association between a mediterranean diet and cardiometabolic disease risk. Nat Med. (2021) 27(2):333–43. 10.1038/s41591-020-01223-3 33574608 PMC8186452

[50] Morrison KE Jašarević E Howard CD Bale TL. It’s the fiber, not the fat: significant effects of dietary challenge on the gut microbiome. Microbiome. (2020) 8(1):15. 10.1186/s40168-020-0791-6 32046785 PMC7014620

[51] Corbin KD Carnero EA Dirks B Igudesman D Yi F Marcus A Host-diet-gut microbiome interactions influence human energy balance: a randomized clinical trial. Nat Commun. (2023) 14(1):3161. 10.1038/s41467-023-38778-x 37258525 PMC10232526

[52] Costabile A Deaville ER Morales AM Gibson GR. Prebiotic potential of a maize-based soluble fibre and impact of dose on the human gut microbiota. PLoS One. (2016) 11(1):e0144457. 10.1371/journal.pone.0144457 26731113 PMC4701468

[53] Deehan EC Yang C Perez-Muñoz ME Nguyen NK Cheng CC Triador L Precision microbiome modulation with discrete dietary fiber structures directs short-chain fatty acid production. Cell Host Microbe. (2020) 27(3):389–404.e6. 10.1016/j.chom.2020.01.006 32004499

[54] Whisner CM Martin BR Nakatsu CH Story JA MacDonald-Clarke CJ McCabe LD Soluble corn fiber increases calcium absorption associated with shifts in the gut microbiome: a randomized dose-response trial in free-living pubertal females. J Nutr. (2016) 146(7):1298–306. 10.3945/jn.115.227256 27281813

[55] Rodriguez CI Isobe K Martiny JBH. Short-term dietary fiber interventions produce consistent gut microbiome responses across studies. mSystems. (2024) 9(6):e0013324. 10.1128/msystems.00133-24 38742890 PMC11237734

[56] Vandeputte D Falony G Vieira-Silva S Wang J Sailer M Theis S Prebiotic inulin-type fructans induce specific changes in the human gut microbiota. Gut. (2017) 66(11):1968–74. 10.1136/gutjnl-2016-313271 28213610 PMC5739857

[57] Baxter NT Schmidt AW Venkataraman A Kim KS Waldron C Schmidt TM. Dynamics of human gut microbiota and short-chain fatty acids in response to dietary interventions with three fermentable fibers. mBio. (2019) 10(1):e02566-18. 10.1128/mBio.02566-18 30696735 PMC6355990

[58] Murga-Garrido SM Hong Q Cross T-WL Hutchison ER Han J Thomas SP Gut microbiome variation modulates the effects of dietary fiber on host metabolism. Microbiome. (2021) 9(1):1–26. 10.1186/s40168-021-01061-6 34016169 PMC8138933

[59] Arifuzzaman M Won TH Yano H Uddin J Emanuel ER Hu E Dietary fiber is a critical determinant of pathologic ILC2 responses and intestinal inflammation. J Exp Med. (2024) 221(5):e20232148. 10.1084/jem.20232148 38506708 PMC10955042

[60] van den Heuvel EG Muys T van Dokkum W Schaafsma G. Oligofructose stimulates calcium absorption in adolescents. Am J Clin Nutr. (1999) 69(3):544–8. 10.1093/ajcn/69.3.544 10075343

[61] van den Heuvel EG Schaafsma G Muys T van Dokkum W. Nondigestible oligosaccharides do not interfere with calcium and nonheme-iron absorption in young, healthy men. Am J Clin Nutr. (1998) 67(3):445–51. 10.1093/ajcn/67.3.445 9497188

[62] Martin-Gallausiaux C Marinelli L Blottière HM Larraufie P Lapaque N. SCFA: mechanisms and functional importance in the gut. Proc Nutr Soc. (2021) 80(1):37–49. 10.1017/S0029665120006916 32238208

[63] Passalacqua G Ciprandi G Canonica GW. The nose-lung interaction in allergic rhinitis and asthma: united airways disease. Curr Opin Allergy Clin Immunol. (2001) 1(1):7–13. 10.1097/00130832-200102000-00003 11964663

[64] Bachert C Luong AU Gevaert P Mullol J Smith SG Silver J The unified airway hypothesis: evidence from specific intervention with anti-IL-5 biologic therapy. J Allergy Clin Immunol Pract. (2023) 11(9):2630–41. 10.1016/j.jaip.2023.05.011 37207831

[65] Chen M Ge Y Lin W Ying H Zhang W Yu X Clinical features and nasal inflammation in asthma and allergic rhinitis. Clin Exp Immunol. (2022) 208(1):25–32. 10.1093/cei/uxac019 35348596 PMC9113297

[66] Ni D Pinget G Santner-Nanan B Tan J Reyes JGA Lai CL Effects of butyrylated high amylose maize starch (HAMSB) as an adjuvant for oral immunotherapy. Allergy. (2025):1–5. 10.1111/all.70161 41258884

[67] Lewis G Wang B Shafiei Jahani P Hurrell BP Banie H Aleman Muench GR Dietary fiber-induced microbial short chain fatty acids suppress ILC2-dependent airway inflammation. Front Immunol. (2019) 10:2051. 10.3389/fimmu.2019.02051 31620118 PMC6760365

