# Supplementary figures and images for: A randomized, double-blind, placebo-controlled trial of soluble corn fiber supplementation for children with asthma
Source: Front Allergy. 2026 Jan 21;6:1707834. doi: 10.3389/falgy.2025.1707834 (PMC12868147; doi:10.3389/falgy.2025.1707834)

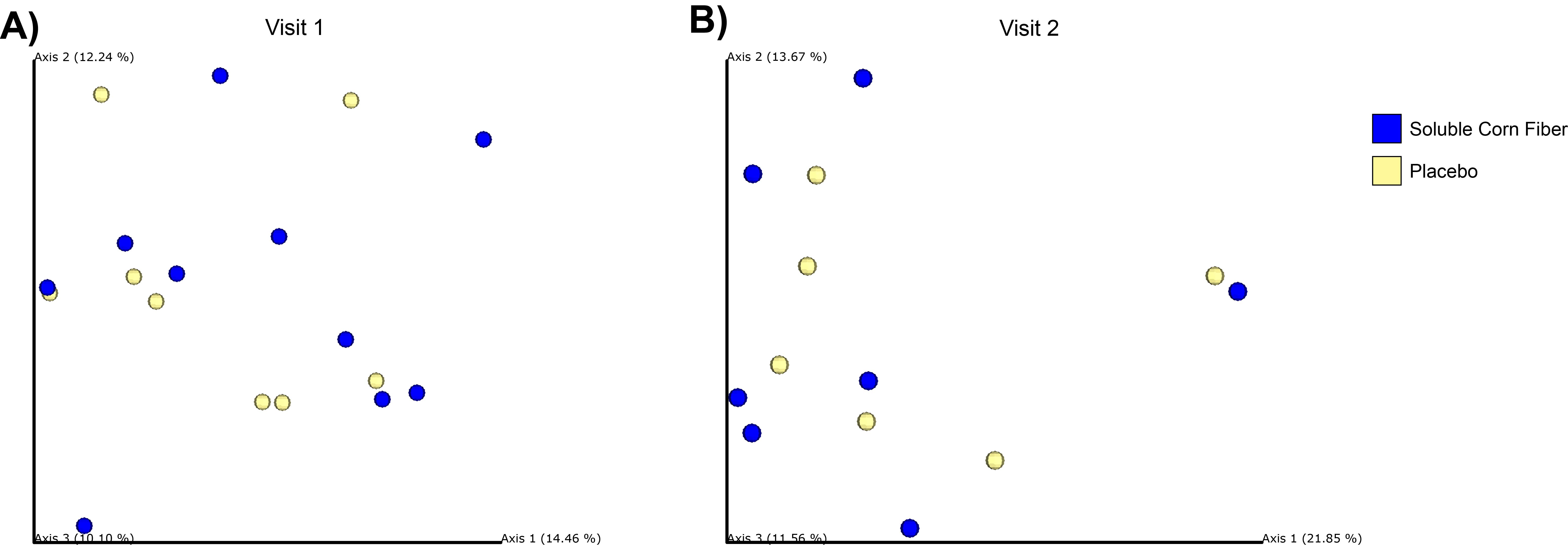

Supplement: Supplementary Figure S1 — No clustering in fecal samples by treatment arm at Visit 1 or Visit 2. (A) PCoA of Unweighted UniFrac of fecal samples at Visit 1 do not show clear patterns of separation between treatment arms. (B) PCoA of Unweighted UniFrac of fecal samples at Visit 2 do not show clear patterns of separation between treatment arms. [file Image1.jpeg]

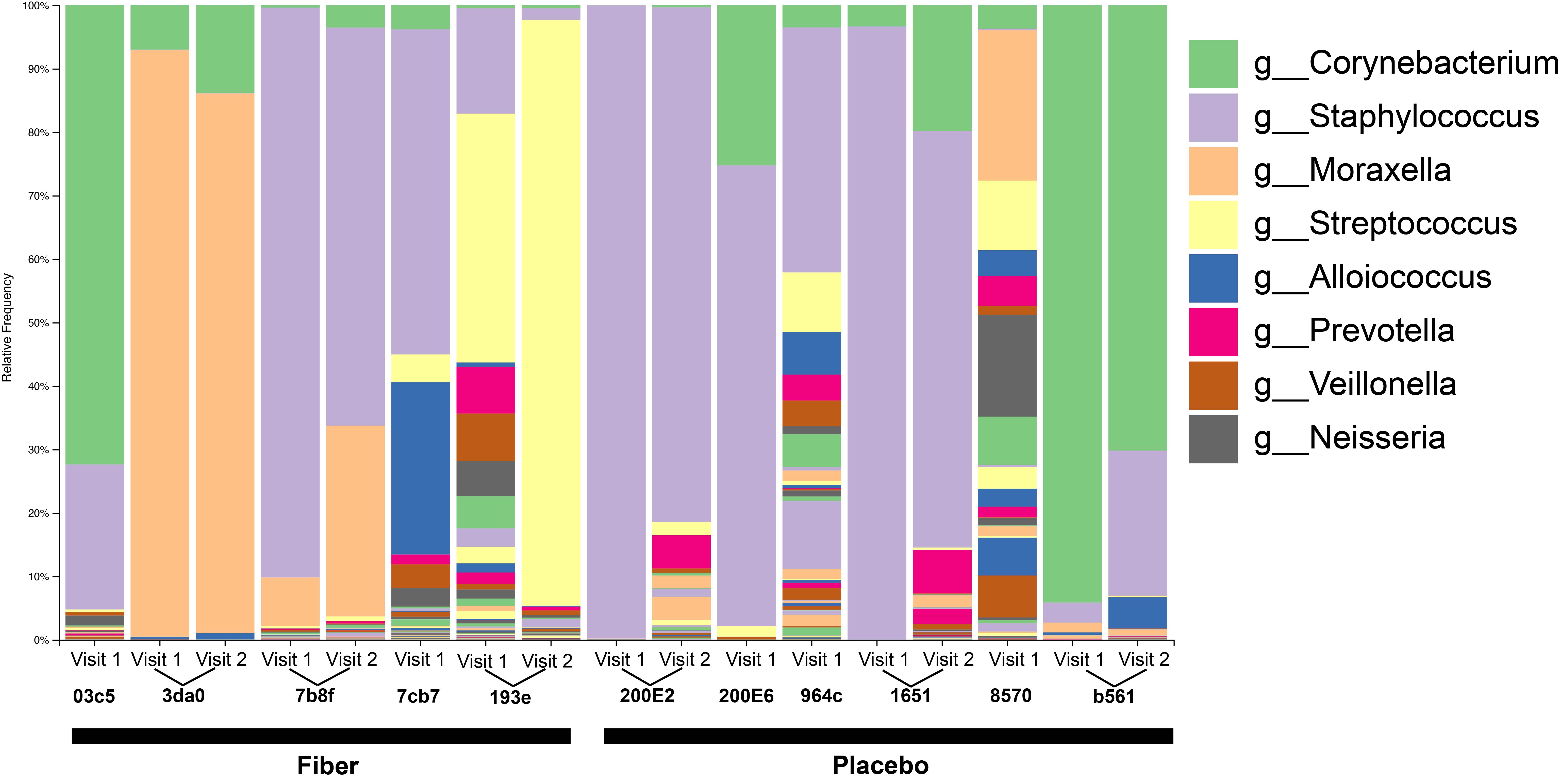

Supplement: Supplementary Figure S2 — Taxonomic bar plot of the nasal microbiome in participants receiving fiber supplement or placebo control. Three participants in the fiber arm and three participants in the placebo arm had paired samples at Visit 1 and Visit 2. Five of 8 participants in the Fiber arm and 6 of 7 participants in the Placebo arm had at least a Visit 1 specimen. In the paired samples, the nasal microbiome did not substantially change after the intervention period nor did the intervention type influence the nasal microbiome composition. [file Image2.jpeg]

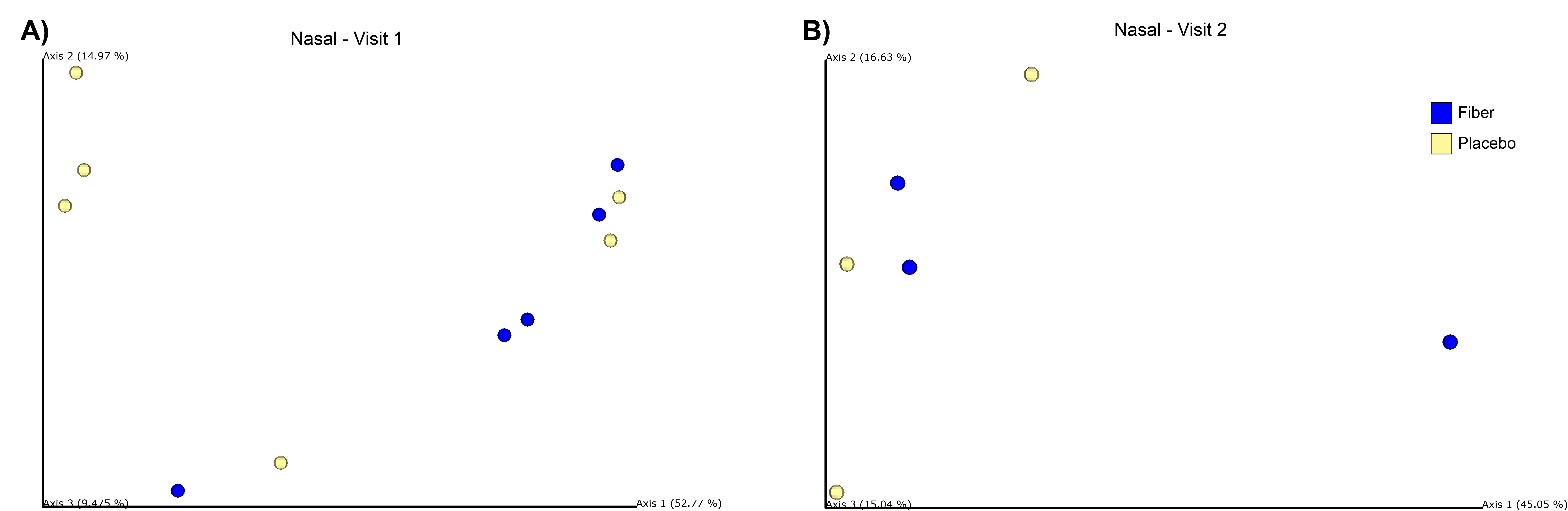

Supplement: Supplementary Figure S3 — No clustering in nasal samples by treatment arm at Visit 1 or Visit 2. (A) PCoA of Unweighted UniFrac of nasal samples at Visit 1 do not show clear patterns of separation between treatment arms. (B) PCoA of Unweighted UniFrac of nasal samples at Visit 2 do not show clear patterns of separation between treatment arms. [file Image3.jpeg]
